# Material Conditions and Well-Being in Old Age: the Role of Contextual Factors at Regional Level

**DOI:** 10.1093/geroni/igaa057.2508

**Published:** 2020-12-16

**Authors:** Michal Myck, Martina Brandt, Claudius Garten, Monika Oczkowska, Alina Schmitz

**Affiliations:** 1 Centre for Economic Analysis, CenEA, Szczecin, Zachodniopomorskie, Poland; 2 TU Dortmund, Dortmund, Nordrhein-Westfalen, Germany; 3 TU Dortmund, Institute for Sociology, Dortmund, Nordrhein-Westfalen, Germany

## Abstract

In our Beethoven project “Age-well” we examine the role of regional contextual factors for the relationship between individual wellbeing and material conditions over time, using a unique combination of individual and regional level longitudinal data. The analyses consider a broad range of regional, community-level indicators for two countries, Germany and Poland, both characterised by rapid population ageing and significant regional variation in the standard of living. These variables, including local indicators of economic conditions and public services, are combined with detailed individual-level information on wellbeing from the Survey of Health, Ageing and Retirement in Europe (SHARE). This data match allows us to study the degree to which regional contexts affect the relationship between individual material conditions and wellbeing in later life. Local public services are shown to mediate the importance of individual level resources for wellbeing confirming an important channel through which public policy can improve welfare of older people.

